# The Use of Passive Smartphone Data to Monitor Anxiety and Depression Among College Students in Real-World Settings: Protocol for a Systematic Review

**DOI:** 10.2196/38785

**Published:** 2022-12-14

**Authors:** Eva Girousse, Nicolas Vuillerme

**Affiliations:** 1 AGEIS Université Grenoble Alpes Grenoble France; 2 LabCom Telecom4Health Orange Labs & Université Grenoble Alpes, CNRS, Inria, Grenoble INP-UGA Grenoble France; 3 Orange Labs Meylan France; 4 Institut Universitaire de France Paris France

**Keywords:** smartphones, anxiety, depression, college students, smartphone, data, monitor, students, systematic review, public health, mental conditions, disorder, strength, limitation

## Abstract

**Background:**

College students are particularly at risk of depression and anxiety. These disorders have a serious impact on public health and affect patients’ daily lives. The potential for using smartphones to monitor these mental conditions, providing passively collected physiological and behavioral data, has been reported among the general population. However, research on the use of passive smartphone data to monitor anxiety and depression among specific populations of college students has never been reviewed.

**Objective:**

This review’s objectives are (1) to provide an overview of the use of passive smartphone data to monitor depression and anxiety among college students, given their specific type of smartphone use and living setting, and (2) to evaluate the different methods used to assess those smartphone data, including their strengths and limitations.

**Methods:**

This review will follow the PRISMA (Preferred Reporting Items for Systematic Reviews and Meta-Analyses) guidelines. Two independent investigators will review English-language, full-text, peer-reviewed papers extracted from PubMed and Web of Science that measure passive smartphone data and levels of depression or anxiety among college students. A preliminary search was conducted in February 2022 as a proof of concept.

**Results:**

Our preliminary search identified 115 original articles, 8 of which met our eligibility criteria. Our planned full study will include an article selection flowchart, tables, and figures representing the main information extracted on the use of passive smartphone data to monitor anxiety and depression among college students.

**Conclusions:**

The planned review will summarize the published research on using passive smartphone data to monitor anxiety and depression among college students. The review aims to better understand whether and how passive smartphone data are associated with indicators of depression and anxiety among college students. This could be valuable in order to provide a digital solution for monitoring mental health issues in this specific population by enabling easier identification and follow-up of the patients.

**Trial Registration:**

PROSPERO CRD42022316263; https://www.crd.york.ac.uk/prospero/display_record.php?RecordID=316263

**International Registered Report Identifier (IRRID):**

DERR1-10.2196/38785

## Introduction

### Background

Depression is recognized as a leading cause of poor health globally [1]. It is defined as a succession of characteristic depressive episodes, with symptoms including pathological sadness, loss of pleasure, and cognitive symptoms. Depression can lead to an increased risk of death, with suicide rates among clinically depressed patients ranging from 5% to 20% [2]. Anxiety disorders are also common in the general population, with 20% of adults having anxiety at one point in their lives. People affected by anxiety disorders feel intense and persistent anxiety without any tangible danger but in a way that affects their daily life [3]. Anxiety disorders are often correlated with depression and precede it in most cases, even if the inverse has also been observed [3]. Both mental disorders should be treated as early as possible to prevent the risk of relapse and aggravation [2,3].

College students experience substantially higher rates of depression than the general population [4], and several studies have shown that anxiety is prevalent among them [5-10]. One study identified several risk factors for anxiety and depression that were directly linked to college, such as tuition fees and the college’s location [6]. Another study noted that regular alcohol consumption—a common behavior among college students—is also a risk factor for anxiety [10]. Mental disorders among college students are a growing concern, and this was especially true during the COVID-19 pandemic. It has been shown that the COVID-19 pandemic led to increases in several psychological disorders among the general population [11], and this result has been confirmed among college students: the prevalence of depression and anxiety greatly increased during the COVID-19 pandemic, rising from 21% and 19%, respectively, before March 1, 2020, to 54% and 37% afterward [12].

The smartphone’s potential to help address the critical and challenging issues of depression and anxiety among college students deserves a careful analysis. Smartphones are now ubiquitous in modern societies, with 6.37 billion smartphone users worldwide in 2021, and the average smartphone user checks their smartphone 63 times a day, according to Bankmycell [13,14]. Smartphones are even more present in the lives of young Europeans aged 16-29 years [15].

This omnipresence of smartphones among college students makes them important features in young people’s mental health. Indeed, smartphones have been studied as both a potential cause of disorders [16] and a tool for interventional treatment [17]. They also enable data collection through passive sensing, which is the collection of environmental and personal data about a user, with minimal interaction and effort on their part. These passive smartphone data are therefore collected continuously with minimal burden on the user, making it suitable for analyzing the user’s mental health. Hence, passive smartphone data collection may provide a clinical opportunity to detect and monitor mental health disorders in the specific population of college students.

The use of passive smartphone data to monitor health and well-being has been reported previously, highlighting the benefits of passive sensing for monitoring mental health, sleep, and sociability, or for fall detection [18,19]. More precisely, passive data from smartphones and wearable devices have been reported as useful in monitoring clinical populations with specific mental health conditions, such as bipolar disorders, schizophrenia, autism, or psychosis [20-23]. Specifically, the use of passive smartphone data for monitoring depressive mood symptoms in people with bipolar disorders has been widely studied [24-32]. Faurholt-Jepsen et al [24,25] found that several features correlate with depressive symptoms, including the number of SMS text messages, the daily average duration of calls, and the amount of screen time. Previous studies also reported the use of machine learning approaches to establish a link between the geolocation of smartphone data [26] or acoustic features from the smartphone’s microphone [27], and depressive mood symptoms in people with bipolar disorders [26,27]. Additionally, the use of passive smartphone data for monitoring depression has been studied both in the nonclinical population and in the clinical population with a diagnosis of depression [33-35]. Concerning this clinical population, these studies interestingly showed significant correlations between depressive symptoms and phone call features [33,34]. While one study involving adolescents (n=13, mean age: 14.93 years) showed a significant negative correlation between depression scores and daily average call duration [34], another study involving adults (n=74, mean age: 44.4 years) showed a significant positive correlation [33]. In the nonclinical population, passive smartphone data has been used to monitor anxiety and depression [21,36-42], as well as mental health–related issues, such as stress, well-being, and loneliness [43-49]. These studies reported a large range of possible features derived from passive smartphone data. Alongside the features cited above for clinical populations, we notably reported the time spent in natural outdoor environments [44], sleep disturbance features [40], and daily time spent running [42].

Using passive smartphone data to monitor mental health encounters several challenges that have been previously pointed out by Trifan et al [18] and Harari et al [50]. First, when passive data is collected through an app, battery use and app design are major challenges. Users expect that the battery will not drop out due to the app running in the background continuously and that the app will be easy to use [18]. Providing health-related feedback may help to ensure user interest and adherence [18]. To conduct ethical research, researchers must ensure transparency toward participants. This transparency can be achieved with an informed consent process, including a starting session explaining what and how data will be collected, and a debriefing interview at the end of the study [50]. Authors also raise the fact that studies involving smartphone data demand attention to privacy and security [18,50].

To the best of our knowledge, no prior work has systematically reviewed the use of passive smartphone data to monitor depression and anxiety among college students. Yet, college students are a specific population, both in the way they use their smartphones and in how they experience anxiety and depression. Because they are more at risk from these mental disorders, we should not miss this potential clinical opportunity to help them use a tool that is in every student’s pocket. By identifying and analyzing relevant studies on the topic, we can improve our understanding of the potential to use passive smartphone data to monitor depression and anxiety among college students. This protocol describes the design and methods for a systematic review of published studies analyzing the use of passive smartphone data to monitor depression and anxiety among college students.

### Objectives

The review aims to better understand whether and how passive smartphone data is associated with indicators of depression and anxiety among college students. To do so, we will identify and synthesize available literature on methods and main findings regarding the use of passive smartphone data to monitor anxiety and depression among college students.

## Methods

### Overview

We will initiate the systematic review by following the Population, Intervention, Comparison, and Outcome worksheet guidelines [51]. We will select, analyze, and report on the relevant studies by following the PRISMA (Preferred Reporting Items for Systematic Reviews and Meta-Analyses) checklist and guidelines [52]. We recently registered this protocol for our systematic review on the International Prospective Register of Systematic Reviews (CRD42022316263).

### Eligibility Criteria

We did not restrict the search to a specific time frame of publication.

The inclusion criteria are as follows:

1. The study participants were college students (in this study, “college students” refer to those who are enrolled in a higher education program, as previously done by Li et al [12] and Lattie et al [53]).
2. The study involved features derived from passive smartphone data. Passive smartphone data refers to data gathered through passive sensing, that is, smartphone-based collection of environmental and personal data about a user, with minimal interaction and effort on their part [18,19,54]. Minimal interaction and effort mean that the user does not need and should not do any input to produce data. For instance, passive smartphone data can be geolocation logs, call logs, text logs, the amount of screen time, and so on.
3. Participants were assessed on a clinically validated rating scale, either self-reported scales or clinical diagnosis scales, used within psychiatry and psychology to screen for depression and anxiety.
4. The study reported measurements quantifying the use of passive smartphone data to monitor anxiety or depression.
5. The study was either a cohort study or a cross-sectional study.

The third criterion is designed to ensure a broad inclusion of studies reporting anxiety and depression symptoms and has been chosen to include both clinical and nonclinical populations. This is not restricted to a specific rating scale in order to reflect the variety of tools used to assess anxiety and depression symptoms in the literature. However, the inclusion of clinically validated rating scales only, such as Generalized Anxiety Disorder-7 [55], Patient Health Questionnaire-9 [56], or Hamilton Depression Rating Scale [57], was chosen to restrict the analysis to commonly used and validated scales, which enables comparison and generalization.

The exclusion criteria are as follows:

1. The study was not published in English.
2. The study was not published following a thorough peer-review process.
3. No full text was available.
4. The study was one of the following publication types: trial protocols, editorials, letters, opinions, case reports, case studies, reviews, or meta-analyses. Books, book chapters, and other gray literature materials (eg, government reports and theses) will not be included.
5. The study involved only data from smartphone-connected wearables.

This last exclusion criterion is designed to avoid including data from tools that are not as ubiquitous as smartphones, either for financial, cultural, or other reasons. Including data gathered by such tools may lead to a selection bias we want to avoid in the first place, in order to get the most representative sample of college students possible. However, the use of smartphone-connected wearables to monitor mental health conditions has previously been studied in clinical and nonclinical populations [20,23,36].

### Data Sources and Search Strategy

Two independent reviewers will search the PubMed and Web of Science electronic databases for published studies meeting the search criteria. No limit to publication dates will be applied. Database-specific search strings will be designed using the Boolean operators “AND” and “OR” in combination with MESH descriptors that might be found in titles or abstracts. The search string will include a combination of terms relating to (1) passive smartphone data and (2) anxiety and depression. The search strings for PubMed and Web of Science were as follows: (“mobile phone location data” OR “mobile phone call data” OR “mobile phone data” OR “cell phone data” OR “cell phone call data” OR “cell phone location data” OR “smartphone location data” OR “smartphone call data” OR “ smartphone data” OR “call detail records”) AND (“depression” OR “affective disorder*” OR “anxiety” OR “anxiety disorder*” OR “mental health” OR “mood disorder*” OR “unipolar” OR “mental disorder*“).

### Study Selection

After the removal of duplicates, 2 independent reviewers will screen the articles for eligibility. The 2 reviewers will go through each article’s title, abstract, and keywords to make a first selection based on our eligibility criteria. If eligible, the full text will be retrieved, and the same reviewers will screen these for eligibility. In cases of disagreement, the 2 reviewers will find a consensus through discussion, and a third reviewer will be consulted should this prove impossible. As a proof of concept, a preliminary search and study selection were conducted in February 2022.

### Data Extraction

The following 5 data sets will be extracted from each article and compared for coherence by the same 2 reviewers: (1) study characteristics, (2) participant characteristics, (3) methods of passive smartphone data collection and measurement, (4) methods of assessment of anxiety and depression, and (5) main findings on the association between passive smartphone data and levels of anxiety or depression among college students. The authors of the study will be contacted in cases of missing or incoherent data. In cases of disagreement between the 2 reviewers, they will attempt to find a consensus through discussion but will consult a third reviewer should this fail.

### Data Synthesis and Analysis

We aim to investigate the use of passive smartphone data to monitor depression and anxiety among college students. We will thus systematically review publications that report any measurements of passive smartphone data and levels of depression or anxiety among college students and that provide any means of using these data to monitor this specific situation. We will report these measurements according to the method used (eg, correlations or regression-based machine learning) and the type of study (cross-sectional or cohort study), with specific attention to the cross-sectional studies, where the causation may be bidirectional. We will compare the results for clinical and nonclinical samples and, notably, verify if the directionality of the association between smartphone features and indicators of depression and anxiety is the same among these two populations. Given that the study will include cross-sectional and cohort studies, no single quality assessment tool is suitable. Hence, the methodological quality of the studies included will be evaluated using a tool created by De Angel and colleagues [36] for a review with a similar scope. The tool combines the Appraisal Tool for Cross-Sectional Studies [58] and the Newcastle–Ottawa Scale for longitudinal studies [59]. Two independent reviewers will analyze the articles for information about the publication of a protocol, the definition of outcomes, evidence of selective reporting, sample descriptions and definitions of eligibility, statistical controls for confounding and multiple comparisons, missing data, representativeness, and justification of the sample size. The publication of a protocol will be scored as 1 if mentioned and 0 if not mentioned. Each question on the quality assessment tool will be scored 0, 1, or 2. A score of 2 will indicate that the study fulfills the assessment criterion, while a score of 0 will indicate that the assessment criterion is not satisfied. A score of 1 will indicate missing information or a lack of precision in the corresponding items. In cases of disagreement between the 2 reviewers, they will attempt to find a consensus through a discussion but will consult a third reviewer should this fail.

### Ethical Considerations

The proposed review will be limited to publicly available materials and information and, therefore, does not require ethical approval. All results will be made available to the public and the scientific community.

## Results

**Figure 1:**
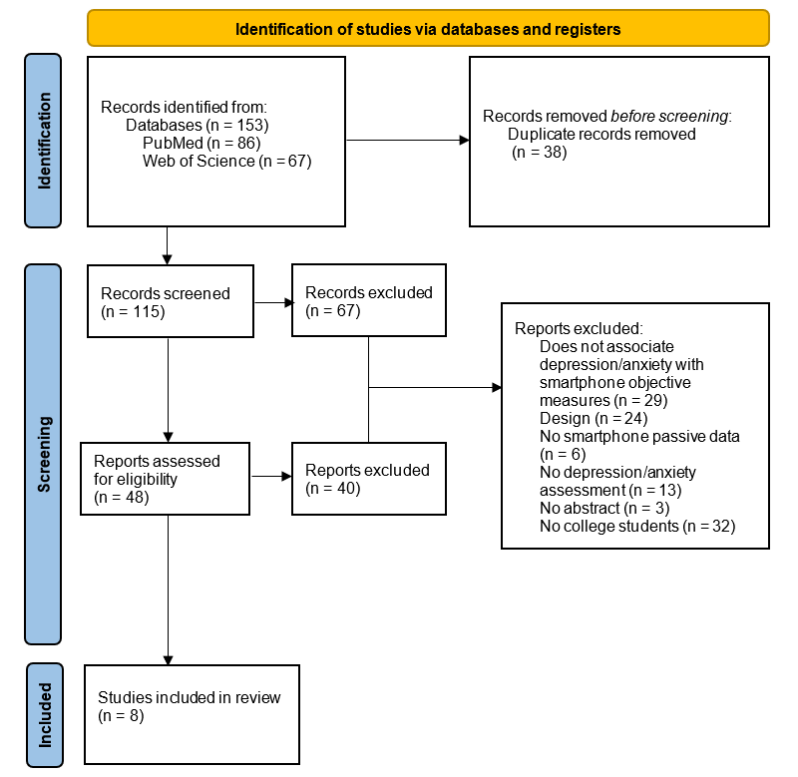
Study selection flowchart. Electronic databases were searched to retrieve relevant studies. This flowchart represents the review’s eligibility criteria.

As a proof of concept, a preliminary search and study selection were conducted in February 2022 and are schematized in Figure 1. We identified 153 records, of which 115 remained after the removal of duplicates. Of these potentially eligible studies, only 8 fully satisfied our inclusion criteria. We are currently going through the process of data extraction and analysis. We will provide a flowchart that summarizes the search strategy, as well as tables and figures presenting the extracted data and the results of the study quality assessment. We intend to make our results available to an international audience through publication in a peer-reviewed journal.

## Discussion

### Expected Findings

We expect that the findings of this systematic review will have the potential to inform researchers and practitioners about how passive smartphone data has been used to monitor depression and anxiety among college students. In particular, since smartphones are an artifact of everyday life, we expect passive smartphone data to act as proxies for anxiety and depression symptoms in our targeted population.

### Comparison to Prior Work

College students are a specific population, both in the way they use their smartphones [60] and in how they experience anxiety and depression [4]. Their living habits are different from the general population, and their smartphone usage is likely to reflect this fact. Yet, previous systematic reviews [18,19,23,36] focused on a general population (ie, no age restriction beyond limiting to 18- to 65-year-olds), and we do not necessarily expect their conclusions to generalize to college students.

### Preliminary Results and Future Steps

Following our study selection process, we found 8 published articles that satisfied our eligibility criteria [42,61-67]. Three articles studied depression only [42,66,67], 1 studied anxiety only [65], and 4 included both depression and anxiety [61-64]. Other mental disorders—or behaviors related to mental disorders—were analyzed in these studies, such as loneliness, disturbed sleep, or stress. Two studies based on the same data sets included 816 participants. The number of participants included in the other 6 studies ranged from 20 to 100. Three studies [61,64,65] examined the use of passive smartphone data to predict mental health. The remaining studies either examined associations between passive smartphone data and mental health indicators [62-65] or aimed to extract the key features from smartphone data that could help identify depression [42]. Our initial examination of these studies suggests that some features based on passive smartphone data, when appropriately used, could indeed help monitor depression or anxiety among college students.

To the best of our current knowledge, no other work has systematically reviewed the use of passive smartphone data to monitor depression and anxiety among college students. We have registered this protocol for our systematic review on the International Prospective Register of Systematic Reviews (CRD42022316263). We will conduct and report our systematic review according to the PRISMA checklist and guidance [52]. Through this process, we aim to design the appropriate research questions and a search strategy able to extract all the relevant studies’ findings. We will provide a synthesis of this, including a flowchart that summarizes the search strategy, as well as tables and figures presenting the extracted data and the results of the study quality assessment. We intend to make our results available to an international audience through publication in a peer-reviewed journal.

### Strengths and News Value

We believe that our observations will help increase knowledge of how passive smartphone data can be associated with indicators of depression and anxiety among college students. We estimate that focusing on college students is crucial since they are a specific population, both in the way they use their smartphones and how they experience anxiety and depression. Therefore, we believe that our observations could provide knowledge on how to use passively collected smartphone data to monitor depression and anxiety among college students. It could prove valuable in order to increase the quality of care by providing better identification and follow-up of these disorders through digital solutions.

### Limitations

We chose to restrict the set of selected articles to those that have used clinically validated tools to measure anxiety and depression. We did not consider loneliness, well-being, and stress levels even though they may be related to anxiety or depression [68-70]. Additionally, our preliminary search has identified only 1 article based on data collected after March 2020. Therefore, the conclusions drawn from this systematic review might not strictly generalize to lockdown periods during the COVID-19 pandemic.

## Abbreviations

PRISMA: Preferred Reporting Items for Systematic Reviews and Meta-Analyses

## Data Availability

No data were generated for this research protocol.
